# Changes in outpatient antibiotic prescribing for acute respiratory illnesses, 2011 to 2018

**DOI:** 10.1017/ash.2021.230

**Published:** 2021-12-17

**Authors:** Laura M. King, Sharon V. Tsay, Lauri A. Hicks, Destani Bizune, Adam L. Hersh, Katherine Fleming-Dutra

**Affiliations:** 1 Division of Healthcare Quality and Promotion, National Center for Emerging Zoonotic and Infectious Diseases, Centers for Disease Control and Prevention, Atlanta, Georgia; 2 Department of Pediatrics, Division of Infectious Diseases, University of Utah, Salt Lake City, Utah

## Abstract

**Objectives:**

To describe acute respiratory illnesses (ARI) visits and antibiotic prescriptions in 2011 and 2018 across outpatient settings to evaluate progress in reducing unnecessary antibiotic prescribing for ARIs.

**Design:**

Cross-sectional study.

**Setting and patients:**

Outpatient medical and pharmacy claims captured in the IBM MarketScan commercial database, a national convenience sample of privately insured individuals aged <65 years.

**Methods:**

We calculated the annual number of ARI visits and visits with oral antibiotic prescriptions per 1,000 enrollees overall and by age category, sex, and setting in 2011 and 2018. We compared these and calculated prevalence rate ratios (PRRs). We adapted existing tiered-diagnosis methodology for *International Classification of Diseases, Tenth Revision, Clinical Modification* (ICD-10-CM) codes.

**Results:**

In our study population, there were 829 ARI visits per 1,000 enrollees in 2011 compared with 760 ARI visits per 1,000 enrollees in 2018. In 2011, 39.3% of ARI visits were associated with ≥1 oral antibiotic prescription versus 36.2% in 2018. In 2018 compared with 2011, overall ARI visits decreased 8% (PRR, 0.92; 99.99% confidence interval [CI], 0.92–0.92), whereas visits with antibiotic prescriptions decreased 16% (PRR, 0.84; 99.99% CI, 0.84–0.85). Visits for antibiotic-inappropriate ARIs decreased by 9% (PRR, 0.91; 99.99% CI, 0.91–0.92), and visits with antibiotic prescriptions for these conditions decreased by 32% (PRR, 0.68; 99.99% CI, 0.67–0.68) from 2011 to 2018.

**Conclusions:**

Both the rate of antibiotic prescriptions per 1,000 enrollees and the percentage of visits with antibiotic prescriptions decreased modestly from 2011 to 2018 in our study population. These decreases were greatest for antibiotic-inappropriate ARIs; however, additional reductions in inappropriate antibiotic prescribing are needed.

Antibiotics are common outpatient medications,^1^ yet they are often prescribed unnecessarily: 28% of antibiotic prescriptions from doctors’ offices and emergency departments in 2014–2015 were unnecessary.^2^ Inappropriate prescribing is even higher for acute respiratory illnesses (ARIs), for which about half of prescriptions are unnecessary.^2,3^

From 2011 to 2018, overall US outpatient antibiotic prescriptions decreased 5%.^4^ However, few studies have examined temporal trends in unnecessary prescribing across outpatient settings for this period. This may be due to challenges created by diagnostic code changes in 2015 when the *International Classification of Diseases, Tenth Revision, Clinical Modification* (ICD-10-CM) replaced ICD-9-CM. Differences between ICD-9-CM and ICD-10-CM codes may complicate comparisons before and after the switch, although codes for ARIs may correspond better than for other diagnoses.^5^ Recent studies have described unnecessary antibiotic prescribing before (2010–2015)^2^ or after (2016) ICD-10-CM implementation.^6^ Additionally, temporal trends in unnecessary prescribing for ARIs have not been assessed in urgent care and retail health settings.

The primary objective of our study was to describe ARI visits and antibiotic prescriptions in 2011 and 2018 across outpatient settings to evaluate progress in reducing unnecessary antibiotic prescribing for ARIs.

## Methods

### Data source and study population

We identified outpatient visits and oral antibiotic prescriptions in the 2011 and 2018 IBM® MarketScan® Commercial Database (IBM® Watson Health™ We defer to the editors on if this is appropriate for ASHE. The MarketScan commercial database contains claims from a convenience sample of individuals with private, employer-sponsored health insurance ≤65 years of age from >300 employers^7^ and has previously been used to describe US outpatient antibiotic prescribing.^6,8–11^

We identified outpatient visits by service date and enrollee number. We included visits made by enrollees with continuous drug and medical coverage for the day of and 3 days after the visit. We excluded claims from settings where antibiotic prescriptions are unlikely: independent laboratories, pharmacies, ambulances, and mass immunization centers. We also excluded visits concurrent with hospitalizations to limit our study to uncomplicated outpatient visits. We categorized age (child 0–17 years and adult 18–64 years) based on median age during enrollment each year. Region (Northeast, Midwest, South, or West) was considered missing if multiple values were present in a year. We categorized setting as office visits, outpatient hospital, urgent care, emergency department, retail health clinic, and other. If claims from multiple settings were present on the same date, we categorized setting as multiple. Because this was a visit-based analysis, enrollees could contribute >1 visit per year.

We identified antibiotics using MarketScan therapeutic class designations: aminoglycosides, β-lactam antibiotics, cephalosporins, chloramphenicol, erythromycin and macrolides, miscellaneous antibiotics, penicillins, quinolones, sulfonamides (excluding sulfasalazine), tetracyclines, and urinary anti-infectives. We additionally included metronidazole and trimethoprim. We included oral antibiotic prescriptions with ≥1 day supply and excluded those marked as refills. If multiple antibiotic classes or agents were dispensed on the same day, we categorized the class or agent as multiple.

We assigned antibiotic prescriptions to the most recent outpatient visit within a 4-day window (the visit date and the subsequent 3 days). As the antibiotic indication could only be assumed based on diagnoses assigned at visits, we excluded prescriptions not linkable to a visit (31% and 28% of all antibiotic prescriptions in 2011 and 2018, respectively). Prescriptions linked to hospital discharges were excluded. Visits associated with multiple prescriptions dispensed on different days within the 4-day post-visit window were counted only once, and antibiotic class was categorized based on the first prescription dispensed.

### Diagnosis assignment

We adapted the ICD-9-CM antibiotic-indication tiered-diagnosis system from Fleming-Dutra et al^3^ for ICD-10-CM codes. ICD-10-CM codes were categorized by condition and tier by author consensus. This system assigns a single diagnosis category per visit based on the diagnosis most likely to result in an antibiotic prescription (see Supplementary Reference Files). The system divides conditions based on systemic antibiotic indication. Tier 1 contains conditions for which antibiotics are almost always indicated (pneumonia, urinary tract infection, miscellaneous bacterial infection). Tier 2 contains conditions for which antibiotics are sometimes indicated (sinusitis, acute otitis media [AOM], pharyngitis, acne, skin and soft tissue infections, gastrointestinal infections). Tier 3 contains conditions for which antibiotics are almost never indicated (antibiotic-inappropriate ARIs: bronchitis and bronchiolitis, asthma and allergy, viral upper respiratory infection [URI], influenza, nonsuppurative otitis media) and other conditions. We categorized bronchitis, bronchiolitis and asthma, allergy visits with additional diagnostic codes for chronic bronchitis (ICD-9-CM: 491.0, 491.1, 491.8, 491.9; ICD-10-CM: J41, J42, J68.0), emphysema (ICD-9-CM: 492.0, 492.8; ICD-10-CM: J43, J98), or chronic obstructive pulmonary disease (COPD; ICD-9-CM: 491.20, 491.21, 491.22, 496; ICD-10-CM: J44.9) as other respiratory conditions. Unlike the original framework, we categorized all codes previously captured as viral pneumonia (tier 3) as pneumonia (tier 1) because viral pneumonia codes were rarely used in outpatient settings. We additionally added 2 new categories: acute exacerbation of COPD (tier 2) and fever (tier 3) to characterize antibiotic prescribing for these conditions. To capture only antibiotic prescribing for fever with no other conditions, visits with tier 3 diagnostic codes in addition to fever were categorized as all other codes not listed elsewhere. For consistency, these changes were made for both ICD-9-CM and ICD-10-CM codes and, where appropriate, ICD-9-CM code categorizations were updated to mirror the ICD-10-CM classifications. We excluded encounter codes (eg, general exam, routine child health exam: ICD-9-CM code V70-V72 and ICD-10-CM code Z0). Final diagnosis and tier were assigned to each visit based on the first-listed, lowest-tier condition. We applied the tiered-diagnosis algorithm to all visits in our study population. Only visits with ARI diagnoses (pneumonia, acute exacerbation of COPD, sinusitis, pharyngitis, AOM, allergy and asthma, bronchitis and bronchiolitis, viral URI, nonsuppurative otitis media, and influenza) from the tiered-diagnosis algorithm were included for further analysis. We limited our study to ARIs to understand visit and antibiotic prescribing changes related to factors other than diagnostic coding.

### Analysis

We calculated the annual number of ARI visits and ARI visits with oral antibiotic prescriptions per 1,000 enrollees overall and by age category, sex, and setting in 2011 and 2018 in our sample. The number of enrollees was calculated by weighting each enrollee based on their enrollment time during the year. For example, enrollees covered for the entire year were weighted as 1 and those covered for 6 months were weighted as 0.5.

We additionally calculated the percentage of ARI visits with antibiotic prescriptions and used a binomial distribution to estimate corresponding confidence intervals (CIs). We compared the percentage of ARI visits with antibiotic prescriptions in 2011 and 2018 using χ^2^ tests of proportion. We calculated prevalence rate ratios (PRRs) and CIs comparing the number of ARI visits and antibiotic prescriptions per 1,000 enrollees in 2011 versus 2018 (referent, 2011), using a Poisson distribution overall and for each strata. Due to the large sample size in our study, we used an α of .0001, similar to previous studies using these data.^11^ All analyses were conducted using SAS version 9.4 software (SAS Institute, Cary, NC).

These data have been determined to be non–human-subjects research; thus, this study was not subject to institutional review board review by the National Center for Emerging and Zoonotic Infectious Diseases human-subjects advisor.

## Results

### Overall ARI visits

In 2011 and 2018 combined, there were 392,619,001 total outpatient visits captured in the MarketScan commercial database that met our inclusion criteria. 11% of these (n = 43,740,650) were associated with ARI diagnoses and were included in our final study population. In 2011, there were 829 ARI visits per 1,000 enrollees, whereas in 2018, there were 760 ARI visits per 1,000 enrollees (Table 1).

### ARI visits with antibiotic prescriptions

In 2011, 39.3% (99.99% CI, 39.3%–39.4%) of ARI visits were associated with ≥1 oral antibiotic prescription versus 36.2% (99.99% CI, 36.2%–36.3%) in 2018 (Table 2). From 2011 to 2018, standardized per 1,000 enrollees, overall ARI visits decreased 8% (PRR, 0.92; 99.99% CI, 0.92–0.92), whereas visits with antibiotic prescriptions decreased 16% (PRR, 0.84; 99.99% CI, 0.84–0.85).

### Visits and antibiotic prescriptions by age group

**Fig. 1.**
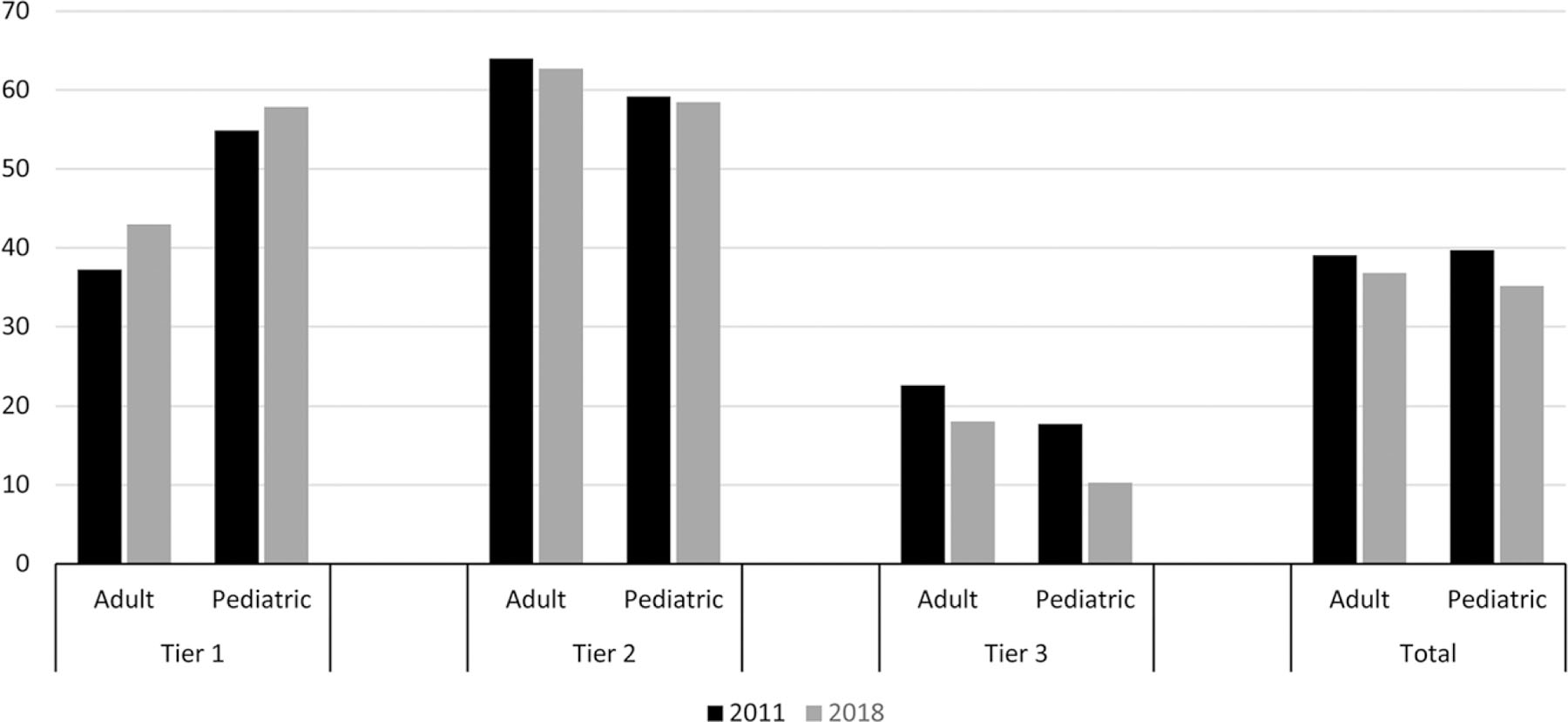
Percentage of acute respiratory illness visits with antibiotic prescriptions by patient age group and antibiotic-indication tier, 2011 and 2018 MarketScan commercial dataset. Outpatient visits with associated oral antibiotic prescriptions by patient age group and antibiotic-indication tier, MarketScan commercial dataset, 2011 and 2018. MarketScan commercial dataset contain data on individuals aged <65 years. Calculated based on median age during MarketScan enrollment in each year.

Children (0–17 years) accounted for 40% of ARI visits in 2011 and 37% of ARI visits in 2018 (Table 1). Per 1,000 enrollees, ARI visits were approximately twice as numerous among children compared with adults. Decreases in both overall ARI visits and ARI visits with antibiotic prescriptions per 1,000 enrollees were greater in children than adults. In children, ARI visits decreased by 10% (PRR, 0.90; 99.99% CI, 0.90–0.90) and ARI visits with antibiotic prescriptions decreased by 20% (PRR, 0.80; 99.99% CI, 0.79–0.80). In adults, ARI visits decreased by 5% (PRR, 0.95; 99.99% CI, 0.95–0.95) and ARI visits with antibiotic prescriptions decreased by 11% (PRR, 0.89; 99.99% CI, 0.89–0.90) (Fig. 1 and Supplementary Table 1). From 2011 to 2018, the percentage of ARI visits with antibiotic prescriptions decreased by 4.5 percentage points in children and 2.3 percentage points in adults.

### Visits and antibiotic prescriptions by tier and condition

Antibiotic-inappropriate ARIs (tier 3) accounted for >50% of all ARI visits in 2011 and 2018 (Table 2). ARI visits per 1,000 enrollees decreased or remained stable for all conditions except influenza and viral URI. Overall, the greatest decrease in visits from 2011 to 2018 was observed for bronchitis and bronchiolitis (36%; PRR, 0.64; 95% CI, 0.64–0.64).

ARIs for which antibiotics are sometimes indicated (tier 2) accounted for majority visits with antibiotic prescriptions in our sample, and prescribing for these conditions decreased 9% (PRR, 0.91; 99.99% CI, 0.91–0.91) from 2011 to 2018 (Table 2). Among these conditions, the percentage of visits with antibiotic prescriptions remained stable or increased for all diagnoses except pharyngitis.

Per 1,000 enrollee prescribing decreased by almost one-third for antibiotic-inappropriate ARIs (tier 3; PRR, 0.68; 99.99% CI, 0.67–0.68), and the percentage of antibiotic-inappropriate ARI visits with antibiotic prescriptions decreased from 20.9% (99.99% CI, 20.9%–20.9%) in 2011 to 15.5% (99.99% CI, 15.4%–15.5%) in 2018.

Antibiotic prescribing per 1,000 enrollees decreased for all conditions except pneumonia and influenza. However, the percentage of influenza visits with antibiotic prescriptions decreased from 15.4% (99.99% CI, 15.1%–15.6%) in 2011 to 8.2% (99.99% CI, 8.0%–8.3%) in 2018. The greatest decreases in per-enrollee antibiotic prescriptions were observed for asthma and allergy and bronchitis and bronchiolitis, conditions for which antibiotics are almost never indicated. Nonetheless, in 2018 >60% of bronchitis and bronchiolitis visits were still associated with antibiotic prescriptions.

Stratified by age, the conditions accounting for the highest proportions of ARI visits with oral outpatient antibiotic prescriptions were sinusitis, pharyngitis, bronchitis, and viral URI in adults and AOM, pharyngitis, and sinusitis in children (Supplementary Table 1).

### Trends by outpatient setting

**Fig. 2.**
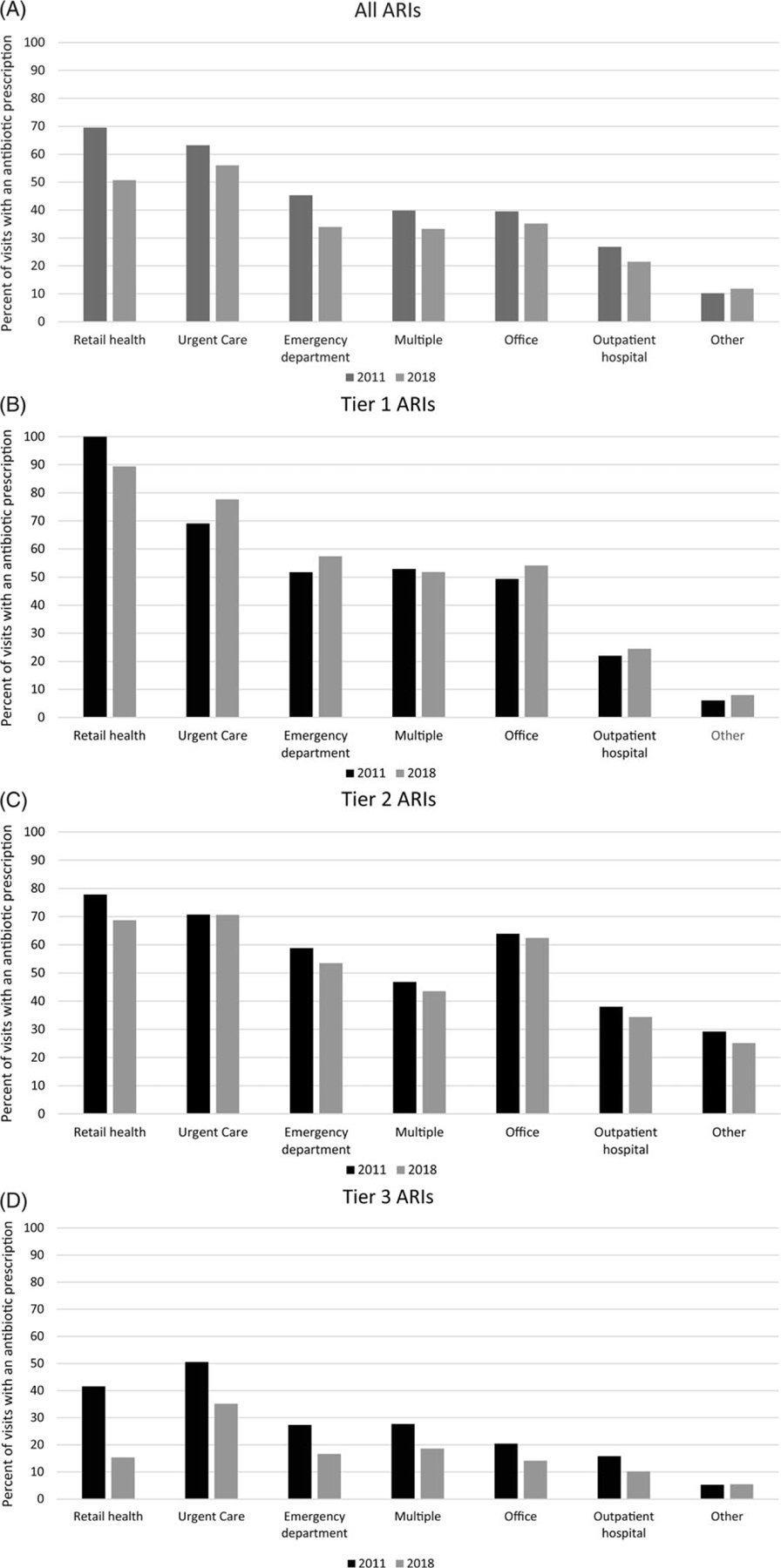
Percent of acute respiratory illness (ARI) visits with associated antibiotic prescription by outpatient setting for (A) all ARIs, (B) ARIs for which antibiotics are almost always indicated, (C) ARIs for which antibiotics are sometimes indicated, (D) Antibiotic-inappropriate ARIs, 2011 and 2018 MarketScan commercial database. Outpatient visits with associated oral antibiotic prescriptions by outpatient setting and antibiotic-indication tier, MarketScan commercial dataset, 2011 and 2018. Other includes telehealth, schools, homeless shelters, Indian Health Services facilities, Tribal facilities, correctional facilities, patient homes, group homes, assisted living facilities, worksites, mobile healthcare units, birthing centers, military treatment facilities, custodial care facilities, hospice, adult living facilities, intermediate care facilities, psychiatric facilities, mental health centers, substance abuse facilities, rehabilitation facilities, dialysis facilities, ambulatory surgery centers, skilled nursing homes, long-term care facilities, inpatient hospital (outpatient services only) and outpatient not elsewhere classified.

More than 80% of ARI visits in both years occurred in physician offices; however, the proportion of visits occurring in urgent care and retail health facilities increased from 2011 to 2018 (Supplementary Table 2). During the study period, the percentage of ARI visits with antibiotic prescriptions decreased across settings except in the other settings category, driven by lower prescribing for antibiotic-inappropriate ARIs across all settings (Fig. 2). Per-visit prescribing rates for ARIs for which antibiotics are sometimes (tier 2) or never (tier 3) indicated were highest in urgent care facilities compared with all other settings. However, the percentage of urgent care visits with antibiotic prescriptions for antibiotic-inappropriate (tier 3) ARIs decreased by ∼15% during the study period from 50.5% (99.99% CI, 50.0%–50.9%) to 35.1% (99.99% CI, 34.8%–35.3%).

### Visits and antibiotic prescriptions by antibiotic class

Penicillins, macrolides, and cephalosporins accounted for the highest proportion of antibiotic prescriptions by class (Supplementary Table 3). From 2011 to 2018, prescriptions decreased across all classes except penicillins, tetracyclines, and other. Decreases were especially notable among fluoroquinolones (51%; PRR 0.49; 99.99% CI, 0.49–0.50), sulfonamides (50%; PRR, 0.50; 99.99% CI, 0.49–0.50), and macrolides (38%; PRR, 0.62; 99.99% CI, 0.62–0.62). Among antibiotic-inappropriate ARIs (tier 3), macrolides were the most common agents, accounting for >50% of all prescriptions in 2011 and 2018. However, between 2011 and 2018, the number of macrolide prescriptions per 1,000 population for these conditions decreased 38% (PRR, 0.62; 99.99% CI, 0.62–0.63).

### Visits and antibiotic prescriptions by region

By region, the South accounted for the largest proportion of visits in both 2011 and 2018 (Table 1). From 2011 to 2018, decreases in both ARI visits and ARI visits with antibiotic prescriptions were observed across regions, with the smallest declines in the South and the largest in the West and Midwest (Supplementary Table 4).

## Discussion

In this study of >40 million outpatient ARI visits in a convenience sample of privately insured individuals under age 65, both the rate of antibiotic prescriptions per 1,000 enrollees and the percentage of visits with antibiotic prescriptions decreased in 2018 compared with 2011. These decreases were greatest for antibiotic-inappropriate ARIs (tier 3), suggesting a decrease in unnecessary antibiotic prescribing. However, in 2018 antibiotics were still prescribed in >15% of visits for antibiotic-inappropriate ARIs. Antibiotic prescriptions for ARIs decreased across outpatient settings, and the percentage of visits with antibiotic prescriptions was highest in urgent care facilities compared with other settings in both 2011 and 2018.

There are likely multiple reasons for fewer ARI visits in 2018 compared with 2011. Care-seeking behaviors for ARIs may have shifted during the study period, potentially related to public health campaigns and previous exposures to delayed prescribing protocols^12^ and non-antibiotic care for some ARIs.^13,14^ Some of the observed decreases are likely related to decreased ARI incidence from uptake of the 13-valent pneumococcal conjugate vaccine,^15,16^ introduced in 2010. Some decreases may also be related to shifts in care to settings not captured in medical claims from which the MarketScan data are sourced (ie, telemedicine). Notably, we observed an increase in influenza visits, likely related to greater influenza incidence in the 2017–2018 and 2018–2019 flu seasons compared with 2010–2011 and 2011–2012.^17^

Conditions for which antibiotics are sometimes appropriate (tier 2) accounted for most visits with antibiotic prescriptions. Among these conditions, the percentage of visits with antibiotic prescriptions decreased only for pharyngitis. Notably, the percentage of pediatric sinusitis and AOM visits with antibiotic prescriptions increased during the study period. Some of the increased proportion of sinusitis and AOM visits with antibiotic prescriptions may be related to the implementation of more stringent diagnostic criteria for AOM^18^ and sinusitis^19,20^ in 2012–2013. Observed decreases in AOM and sinusitis visits with concurrent increases in viral URI suggest that cases that may previously have been diagnosed as AOM or sinusitis but not treated with antibiotics may have been assigned viral URI diagnoses instead.

The greatest decrease in antibiotic prescribing occurred in visits for antibiotic-inappropriate (tier 3) ARIs. The decrease in all ARI visits and antibiotic prescriptions suggests that decreases in prescriptions for antibiotic-inappropriate ARIs reflect true improvements in prescribing rather than just changes in diagnostic patterns. This decrease is at least partially due to improvements in reducing unnecessary antibiotic prescriptions, potentially the result of outpatient antibiotic stewardship efforts, such as the Centers for Disease Control and Prevention Core Elements for Outpatient Antibiotic Stewardship.^21^ However, opportunities to reduce antibiotic prescribing for antibiotic-inappropriate ARIs remain. For example, although antibiotic prescribing for bronchitis and bronchiolitis decreased during the study period, in 2018, >60% of visits for these conditions were still associated with antibiotic prescriptions.

By setting, physicians’ offices accounted for >80% of ARI-related visits in both 2011 and 2018. Even so, we observed an increase in visits to emergency departments, urgent care facilities, and retail health clinics in 2018 compared with 2011. This shift is likely related to the changing landscape of outpatient healthcare with more low-acuity, outpatient visits presenting to urgent care and retail health settings.^22,23^ Shifts in outpatient care setting might explain discrepancies observed in visit trends between our study and a national study including only physician offices and EDs: the study of visits to physician offices and EDs found a 25% decrease in ARI visits from 2010–2011 to 2014–2015,^2^ compared with only an 8% decrease in ARI visits in our study population from 2011 to 2018.

In both 2011 and 2018, the highest proportion of visits with antibiotic prescriptions for antibiotic-inappropriate (tier 3) ARIs was in urgent care facilities, congruent with a previous study using these same data.^9^ Although inappropriate prescribing was highest in urgent care settings for these conditions, we observed a marked decrease in unnecessary prescribing for antibiotic-inappropriate ARI visits, from 50% in 2011 to 35% in 2018, potentially reflecting the success of antibiotic stewardship efforts in urgent care in recent years.^24–26^

We observed greater decreases in antibiotic prescribing to children compared with adults and marked decreases in fluoroquinolone and macrolide prescribing for ARIs, mirroring national antibiotic prescription trends.^2,4^ Differential improvement in prescribing to children may be a result of targeted stewardship efforts in this population, pneumococcal vaccination, and a culture of prescribing improvement among pediatricians.^27^ Decreased fluoroquinolone and macrolide prescribing likely reflects greater awareness of the potential harms of these drugs.^28–30^ Despite decreases in macrolides, azithromycin remained the most commonly prescribed antibiotic for antibiotic-inappropriate ARIs.

Our study had several limitations. First, our study was conducted in a convenience sample of commercially insured individuals aged <65 years and may not be generalizable to other populations. Second, our analysis used diagnostic codes from claims to assign a single diagnosis to each visit and we had no additional clinical information. Third, diagnostic codes and classifications vary between ICD-9-CM to ICD-10-CM; therefore, some changes observed in our study may reflect coding changes versus true shifts in ARI visits and antibiotic prescribing. However, codes for ARIs may correspond better between the 2 schemes (ie, more one-to-one and fewer approximate, one-to-multiple, and multiple-to-one mappings) than for other conditions, so coding changes are likely minor factors in the overall ARI trends observed in our study. Fourth, our visit–prescription linking methodology may have misattributed some prescriptions to visits. Finally, we only examined 2 years (2011 and 2018), so we could not evaluate trends that occurred between those years. However, national outpatient antibiotic prescriptions showed consistent decreases during this period.^1^ A strength of this study was the development and implementation of an antibiotic-indication tiered-diagnosis scheme for ICD-10-CM codes in a large sample capturing visits across outpatient settings.

In conclusion, in this study of >40 million ARI visits using adapted tiered-diagnosis schemes for both ICD-9-CM and ICD-10-CM, we observed a decrease in overall antibiotic prescribing for ARIs driven by decreases in prescribing for antibiotic-inappropriate ARIs and pharyngitis in 2018 compared with 2011. Our findings support the hypothesis that observed decreases in antibiotic prescriptions nationally during this period^4^ are, at least in part, due to reductions in inappropriate prescribing, potentially reflecting stewardship efforts. Although reductions in inappropriate prescribing are encouraging, the decrease observed from 2011 to 2018 was modest. In 2018 inappropriate antibiotic prescriptions still accounted for 63 prescriptions per 1,000 enrollees. Additional focus on outpatient antibiotic stewardship is needed to improve outpatient antibiotic prescribing for ARIs. The ICD-10-CM tiered-diagnosis categorization developed for this study may assist in future efforts to track and improve outpatient antibiotic use.

## Supplementary Material

ICD10 Excel Sheet

Supplementary Table

## Figures and Tables

**Table 1.** Characteristics of Visits for Acute Respiratory Illnesses, MarketScan Commercial Dataset, 2011 and 2018

Characteristic	2011		2018	
	Visits, No. (%)^a^	Visits per 1,000 Enrollees^b^	Visits, No. (%)^a^	Visits per 1,000 Enrollees^b^
All Visits	27,502,268 (100)	829	16,238,382 (100)	760
**Patient age category** ^c^				
Child, 0–17 y	11,015,983 (40)	1,351	5,967,469 (37)	1,214
Adult, 18–64 y	16,486,285 (60)	659	10,270,913 (63)	624
**Patient sex**				
Male	11,884,662 (43)	739	6,974,676 (43)	669
Female	15,617,606 (57)	914	9,263,706 (57)	846
**Region**				
Northeast	4,510,203 (17)	843	2,879,522 (18)	780
Midwest	6,531,042 (24)	812	3,150,532 (20)	693
South	11,773,559 (44)	910	8,019,293 (50)	867
West	3,987,384 (15)	660	2,075,827 (13)	563
**Outpatient setting**				
Office	23,988,158 (87)	723	13,113,034 (81)	614
Outpatient hospital	718,532 (3)	22	415,184 (3)	19
Emergency department	172,895 (1)	5	134,620 (1)	6
Urgent care	521,509 (2)	16	1,333,589 (8)	62
Retail health clinic	226 (0)	0	39,611 (0)	2
Multiple^d^	1,788,050 (7)	54	1,007,901 (6)	47
Other^e^	263,234 (1)	8	167,806 (1)	8

a Numbers may not sum to total due to missing values and percents may not sum to 100 due to rounding.

b Calculated as no. visits/average no. enrollees ×1,000.

c MarketScan commercial dataset contain data on individuals aged <65 y. Calculated based on median age during MarketScan enrollment in each year.

d Setting was categorized as multiple if outpatient services were performed at >1 type of outpatient setting on the same day for one individual or if outpatient services occurred during a hospital stay.

e Other includes telehealth, schools, homeless shelters, Indian Health Services facilities, tribal facilities, correctional facilities, patient homes, group homes, assisted living facilities, worksites, mobile healthcare units, birthing centers, military treatment facilities, custodial care facilities, hospice, adult living facilities, intermediate care facilities, psychiatric facilities, mental health centers, substance abuse facilities, rehabilitation facilities, dialysis facilities, ambulatory surgery centers, skilled nursing homes, long-term care facilities, inpatient hospital (outpatient services only) and outpatient not elsewhere classified.

**Table 2.** Visits and Antibiotic Prescriptions for Acute Respiratory Illnesses by Diagnosis, MarketScan Commercial Dataset, 2011 and 2018

Diagnosis	2011			2018			Comparison of 2011 Versus 2018		
	Visits per 1,000 Enrollees, No. (%)^a^	Visits With Antibiotic Prescription per 1,000 enrollees, No. (%)^b^	Visits with Antibiotic Prescription, % (99.99% CI)^c^	Visits per 1,000 enrollees, No. (%)^a^	Visits with Antibiotic Prescription per 1,000 Enrollees, No. (%)^b^	Visits With Antibiotic Prescription, % (99.99% CI)^c^	*P* Value for χ^2^ tests for Proportion of Visits With Antibiotic Prescription^d^	Prevalence Rate Ratio for No. Visits per 1,000 Enrollees (99.99% CI)^e^	Prevalence Rate Ratio for With Antibiotic Prescription 1,000 Enrollees (99.99%
**All ages**									
**Total**	**829** **(100)**	**326** **(100)**	**39.3** **(39.3–39.4)**	**760** **(100)**	**275** **(100)**	**36.2** **(36.2–36.3)**	**<.0001**	**0.92** **(0.92–0.92)**	**0.84** **(0.84–0.85)**
**Antibiotics almost always indicated (tier 1)**	**21** **(2.5)**	**9** **(2.8)**	**44.1** **(43.9–44.4)**	**19** **(2.5)**	**9** **(3.4)**	**47.9** **(47.6–48.2)**	**<.0001**	**0.93** **(0.92–0.93)**	**1.01** **(0.99–1.02)**
Pneumonia	21 (2.5)	9 (2.8)	44.1 (43.9–44.4)	19 (2.5)	9 (3.4)	47.9 (47.6–48.2)	<.0001	0.93 (0.92–0.93)	1.01 (0.99–1.02)
**Antibiotics sometimes indicated (tier 2)**	**362** **(43.7)**	**224 (68.6)**	**61.7** **(61.7–61.8)**	**333** **(43.8)**	**203** **(73.7)**	**60.9** **(60.9–61.0)**	**<.0001**	**0.92** **(0.92–0.92)**	**0.91** **(0.91–0.91)**
Acute exacerbation of COPD	3 (0.4)	1 (0.3)	31.2 (30.7–31.8)	3 (0.5)	1 (0.4)	31.2 (30.5–31.9)	0.9482	0.99 (0.97–1.00)	0.99 (0.95–1.02)
Pharyngitis	137 (16.5)	70 (21.5)	51.3 (51.2–51.4)	136 (17.9)	63 (23.0)	46.7 (46.5–46.8)	<.0001	0.99 (0.99–1.00)	0.90 (0.90–0.91)
Sinusitis	148 (17.9)	103 (31.5)	69.4 (69.3–69.5)	130 (17.1)	91 (33.2)	70.4 (70.3–70.5)	<.0001	0.88 (0.87–0.88)	0.89 (0.89–0.89)
Acute otitis media	74 (8.9)	50 (15.2)	67.1 (67.0–67.2)	64 (8.4)	47 (17.1)	73.5 (73.4–73.7)	<.0001	0.87 (0.86–0.87)	0.95 (0.94–0.95)
**Antibiotics almost never indicated (tier 3)**	**446** **(53.8)**	**93** **(28.6)**	**20.9** **(20.9–20.9)**	**408** **(53.6)**	**63** **(22.9)**	**15.5** **(15.4–15.5)**	**<.0001**	**0.91** **(0.91–0.92)**	**0.68** **(0.67–0.68)**
Asthma, allergy^f^	240 (29.0)	11 (3.5)	4.7 (4.7–4.7)	206 (27.1)	6 (2.1)	2.8 (2.8–2.8)	<.0001	0.86 (0.86–0.86)	0.51 (0.50–0.51)
Bronchitis, bronchiolitis^f^	55 (6.6)	36 (11.2)	66.0 (65.9–66.2)	35 (4.6)	21 (7.7)	60.1 (59.9–60.3)	<.0001	0.64 (0.63–0.64)	0.58 (0.58–0.58)
Influenza	9 (1.1)	1 (0.4)	15.4 (15.1–15.6)	25 (3.3)	2 (0.7)	8.2 (8.0–8.3)	<.0001	2.87 (2.84–2.90)	1.53 (1.49–1.57)
Non-suppurative otitis media	25 (3.0)	6 (1.9)	24.9 (24.8–25.1)	23 (3.0)	6 (2.2)	27.0 (26.8–27.3)	<.0001	0.91 (0.91–0.92)	0.99 (0.98–1.0)
Viral upper respiratory infection	117 (14.1)	38 (11.7)	32.4 (32.3–32.5)	119 (15.7)	28 (10.2)	23.6 (23.5–23.7)	<.0001	1.02 (1.01–1.02)	0.74 (0.73–0.74)

Note. CI, confidence interval; COPD, chronic obstructive pulmonary disease.

a Calculated as no. visits/average no. enrollees ×1,000.

b Calculated as no. visits with an associated oral antibiotic prescription within a 4-day postvisit window/average no. enrollees × 1,000.

c 99.99% CI estimated using a binomial distribution.

d P value for χ^2^ test comparing percentage of visits with an antibiotic dispensed or administered in 2011 versus 2018, α = .0001.

e Referent is 2011, therefore a prevalence rate ratio of 0.80 indicates that the rate in 2018 was 20% lower than the rate in 2011.

f Visits with an asthma, allergy or bronchitis, bronchiolitis code that had additional codes for chronic bronchitis (ICD-9-CM: 491.0, 491.1, 491.8, 491.9; ICD-10-CM: J41, J42, J68.0), emphysema (ICD-9-CM: 492.0, 492.8; ICD-10-CM: J43, J98), or chronic
