# Supplementary material for: Changes in outpatient antibiotic prescribing for acute respiratory illnesses, 2011 to 2018
Source: Antimicrob Steward Healthc Epidemiol. 2021 Dec 17;1(1):e66. doi: 10.1017/ash.2021.230 (PMC9345578; doi:10.1017/ash.2021.230)
Supplement: Supplementary file 1 [file ashsup.zip › S2732494X21002308sup001.pdf]

Supplemental Tables

Supplemental Table 1. Visits and antibiotic prescriptions for acute respiratory illnesses by age category and diagnosis, MarketScan Commercial Dataset, 2011 and 2018

	2011			2018			Comparison of 2011 versus 2018		
**Diagnosis**	**No. visits per 1000 enrollees (%)[A]**	**No. visits with antibiotic prescrip-tions per 1000 enrollees[B] (%)**	**Percent of visits with antibiotic prescrip-tion (99.99% CI)[C]**	**No. visits per 1000 enrollees (%)[A]**	**No. visits with antibiotic prescrip-tions per 1000 enrollees[B] (%)**	**Percent of visits with antibiotic prescrip-tion (99.99% CI)[C]**	**p-value for chi-square tests for proportion of visits with antibiotic prescrip-tion[D]**	**Prevalence rate ratio for no. visits per 1000 enrollees (99.99% CI)[E]**	**Prevalence rate ratio for no. visits with antibiotic prescrip-tions per 1000 enrollees (99.99% CI)[E]**
**Children (0-17 years)[F]**									
**Total**	**1351 (100.0)**	**537 (100.0)**	**39.7 (39.7-39.8)**	**1214 (100.0)**	**427 (100.0)**	**35.2 (35.1-35.2)**	**<.0001**	**0.90 (0.90-0.90)**	**0.80 (0.79-0.80)**
**Antibiotics almost always indicated**	**33 (2.5)**	**18 (3.4)**	**54.9 (54.5-55.2)**	**28 (2.3)**	**16 (3.8)**	**57.8 (57.3-58.4)**	**<.0001**	**0.84 (0.82-0.85)**	**0.88 (0.87-0.90)**
Pneumonia	33 (2.5)	18 (3.4)	54.9 (54.5-55.2)	28 (2.3)	16 (3.8)	57.8 (57.3-58.4)	<.0001	0.84 (0.82-0.85)	0.88 (0.87-0.90)

	2011			2018			Comparison of 2011 versus 2018		
**Diagnosis**	**No. visits per 1000 enrollees (%)[A]**	**No. visits with antibiotic prescrip-tions per 1000 enrollees[B] (%)**	**Percent of visits with antibiotic prescrip-tion (99.99% CI)[C]**	**No. visits per 1000 enrollees (%)[A]**	**No. visits with antibiotic prescrip-tions per 1000 enrollees[B] (%)**	**Percent of visits with antibiotic prescrip-tion (99.99% CI)[C]**	**p-value for chi-square tests for proportion of visits with antibiotic prescrip-tion[D]**	**Prevalence rate ratio for no. visits per 1000 enrollees (99.99% CI)[E]**	**Prevalence rate ratio for no. visits with antibiotic prescrip-tions per 1000 enrollees (99.99% CI)[E]**
**Antibiotics sometimes indicated**	**687 (50.9)**	**407 (75.8)**	**59.2 (59.1-59.3)**	**600 (49.4)**	**351 (82.1)**	**58.4 (58.3-58.6)**	**<.0001**	**0.87 (0.87-0.88)**	**0.86 (0.86-0.87)**
Pharyngitis	321 (23.8)	152 (28.4)	47.4 (47.3-47.5)	302 (24.9)	130 (30.5)	43.2 (43.0-43.3)	<.0001	0.94 (0.94-0.94)	0.86 (0.85-0.86)
Sinusitis	124 (9.2)	93 (17.4)	75.3 (75.1-75.4)	85 (7.0)	66 (15.5)	78.1 (77.8-78.3)	<.0001	0.68 (0.68-0.69)	0.71 (0.70-0.71)
Acute otitis media	241 (17.8)	161 (30.0)	66.7 (66.8-66.8)	213 (17.6)	154 (36.2)	72.3 (72.1-72.5)	<.0001	0.89 (0.88-0.89)	0.96 (0.95-0.97)
**Antibiotics almost never indicated**	**631 (46.7)**	**112 (20.8)**	**17.7 (17.7-17.8)**	**586 (48.3)**	**60 (14.1)**	**10.3 (10.2-10.3)**	**<.0001**	**0.93 (0.93-0.93)**	**0.54 (0.53-0.54)**
Asthma, allergy[G]	164 (19.6)	14 (2.6)	5.2 (5.2-5.3)	235 (19.4)	5 (1.1)	2.1 (2.0-2.1)	<.0001	0.89 (0.89-0.89)	0.35 (0.34-0.36)

Diagnosis	2011: No. visits per 1000 enrollees (%)[A]	2011: No. visits with antibiotic prescrip-tions per 1000 enrollees[B] (%)	2011: Percent of visits with antibiotic prescrip-tion (99.99% CI)[C]	2018: No. visits per 1000 enrollees (%)[A]	2018: No. visits with antibiotic prescrip-tions per 1000 enrollees[B] (%)	2018: Percent of visits with antibiotic prescrip-tion (99.99% CI)[C]	Comparison of 2011 versus 2018: p-value for chi-square tests for proportion of visits with antibiotic prescrip-tion[D]	Comparison of 2011 versus 2018: Prevalence rate ratio for no. visits per 1000 enrollees (99.99% CI)[E]	Comparison of 2011 versus 2018: Prevalence rate ratio for no. visits with antibiotic prescrip-tions per 1000 enrollees (99.99% CI)[E]
Bronchitis, bronchiolitis[G]	61 (4.5)	34 (6.3)	55.7 (55.4-56.0)	35 (2.8)	14 (3.3)	41.1 (40.7-41.6)	<.0001	0.57 (0.56-0.57)	0.42 (0.41-0.43)
Influenza	17 (1.2)	2 (0.4)	11.3 (11.0-11.6)	42 (3.5)	2 (0.5)	4.8 (4.6-5.0)	<.0001	2.51 (2.48-2.54)	1.07 (1.01-1.12)
Non-suppurative otitis media	63 (4.7)	17 (3.1)	26.5 (26.3-26.8)	57 (4.7)	16 (3.8)	28.9 (28.6-29.3)	<.0001	0.90 (0.89-0.91)	0.98 (0.96-1.00)
Viral upper respiratory infection	226 (16.7)	46 (8.5)	20.2 (20.0-20.3)	218 (17.9)	23 (5.3)	10.4 (10.3-10.5)	<.0001	0.96 (0.96-0.97)	0.50 (0.49-0.51)
**Adults (18-64 years)** [F]									
**Total**	**659 (100)**	**257 (100)**	**39.1 (39.0-39.1)**	**624 (100)**	**230 (100)**	**36.8 (36.7-36.9)**	**<.0001**	**0.95 (0.95-0.95)**	**0.89 (0.89-0.90)**
**Antibiotics almost always indicated**	**17 (2.6)**	**6 (2.4)**	**37.2 (36.9-37.5)**	**17 (2.7)**	**7 (3.1)**	**42.9 (42.6-43.3)**	**<.0001**	**1.00 (0.99-1.01)**	**1.15 (1.14-1.17)**

	2011			2018			Comparison of 2011 versus 2018		
**Diagnosis**	**No. visits per 1000 enrollees (%)[A]**	**No. visits with antibiotic prescrip-tions per 1000 enrollees[B] (%)**	**Percent of visits with antibiotic prescrip-tion (99.99% CI)[C]**	**No. visits per 1000 enrollees (%)[A]**	**No. visits with antibiotic prescrip-tions per 1000 enrollees[B] (%)**	**Percent of visits with antibiotic prescrip-tion (99.99% CI)[C]**	**p-value for chi-square tests for proportion of visits with antibiotic prescrip-tion[D]**	**Prevalence rate ratio for no. visits per 1000 enrollees (99.99% CI)[E]**	**Prevalence rate ratio for no. visits with antibiotic prescrip-tions per 1000 enrollees (99.99% CI)[E]**
Pneumonia	17 (2.6)	6 (2.4)	37.2 (36.9-37.5)	17 (2.7)	7 (3.1)	42.9 (42.6-43.3)	<.0001	1.00 (0.99-1.01)	1.15 (1.14-1.17)
**Antibiotics sometimes indicated**	**256 (38.9)**	**164 (63.7)**	**64.0 (63.9-64.0)**	**253 (40.6)**	**159 (69.1)**	**62.7 (62.6-62.8)**	**<.0001**	**0.99 (0.99-0.99)**	**0.97 (0.96-0.97)**
Acute exacerbation of COPD	4 (0.7)	1 (0.5)	31.0 (30.4-31.5)	4 (0.7)	1 (0.6)	31.4 (30.8-32.1)	0.0349	1.01 (0.99-1.03)	1.03 (0.99-1.06)
Pharyngitis	77 (11.6)	43 (16.9)	56.7 (56.5-56.8)	86 (13.8)	43 (18.9)	50.3 (50.1-50.5)	<.0001	1.12 (1.12-1.13)	1.0 (0.99-1.0)
Sinusitis	156 (23.7)	106 (41.2)	67.9 (67.8-68.0)	143 (23.0)	99 (43.1)	69.1 (68.9-69.2)	<.0001	0.92 (0.92-0.92)	0.94 (0.93-0.94)
Acute otitis media	19 (3.0)	13 (5.2)	68.5 (68.3-68.8)	19 (3.1)	15 (6.5)	77.6 (77.3-77.9)	<.0001	0.99 (0.98-1.0)	1.12 (1.11-1.14)

	2011			2018			Comparison of 2011 versus 2018		
**Diagnosis**	**No. visits per 1000 enrollees (%)[A]**	**No. visits with antibiotic prescrip-tions per 1000 enrollees[B] (%)**	**Percent of visits with antibiotic prescrip-tion (99.99% CI)[C]**	**No. visits per 1000 enrollees (%)[A]**	**No. visits with antibiotic prescrip-tions per 1000 enrollees[B] (%)**	**Percent of visits with antibiotic prescrip-tion (99.99% CI)[C]**	**p-value for chi-square tests for proportion of visits with antibiotic prescrip-tion[D]**	**Prevalence rate ratio for no. visits per 1000 enrollees (99.99% CI)[E]**	**Prevalence rate ratio for no. visits with antibiotic prescrip-tions per 1000 enrollees (99.99% CI)[E]**
**Antibiotics almost never indicated**	**385 (58.5)**	**87 (33.9)**	**22.6 (22.5-22.6)**	**354 (56.8)**	**64 (27.8)**	**18.0 (18.0-18.1)**	**<.0001**	**0.92 (0.92-0.92)**	**0.73 (0.73-0.74)**
Asthma, allergy[G]	232 (35.2)	11 (4.1)	4.5 (4.5-4.6)	197 (31.6)	6 (2.6)	3.0 (3.0-3.1)	<.0001	0.85 (0.85-0.85)	0.57 (0.56-0.58)
Bronchitis, bronchiolitis[G]	53 (8.1)	37 (14.4)	69.9 (69.7-70.0)	35 (5.7)	23 (10.1)	65.6 (65.4-65.9)	<.0001	0.66 (0.66-0.67)	0.62 (0.62-0.63)
Influenza	6 (0.9)	1 (0.5)	19.0 (18.6-19.4)	20 (3.2)	2 (0.9)	10.3 (10.1-10.5)	<.0001	3.27 (3.23-3.31)	1.77 (1.72-1.83)
Non-suppurative otitis media	12 (1.9)	3 (1.1)	22.3 (22.0-22.6)	12 (2.0)	3 (1.3)	24.4 (24.1-24.8)	<.0001	1.01 (1.00-1.02)	1.11 (1.09-1.14)
Viral upper respiratory infection	82 (12.4)	36 (13.8)	43.4 (43.3-43.6)	90 (14.4)	30 (12.9)	33.1 (33.0-33.3)	<.0001	1.10 (1.09-1.10)	0.84 (0.83-0.84)

No. – Number; CI – Confidence interval; COPD – Chronic Obstructive Pulmonary Disease

[A] No. visits per 1000 enrollees calculated as no. visits/average no. enrollees x 1000. Percents may not sum to 100 due to rounding.

[B] No. visits with antibiotic prescriptions per 1000 enrollees calculated as no. visits with an associated oral antibiotic prescription within a 4-day post-visit window/average no. enrollees x 1000. Percents may not sum to 100 due to rounding.

[C] 99.99% CI estimated using a binomial distribution.

[D] P-value for chi-square test comparing percent of visits with an antibiotic dispensed or administered in 2011 versus 2018, alpha = 0.0001.

[E] Referent is 2011, therefore a prevalence rate ratio of 0.80 indicates that the rate in 2018 was 20% lower than the rate in 2011.

[F] MarketScan Commercial datasets contain data on individuals aged <65 years. Calculated based on median age during MarketScan enrollment in each year.

[G] Visits with an asthma, allergy or bronchitis, bronchiolitis code that had additional codes for chronic bronchitis (ICD-9-CM: 491.0, 491.1, 491.8, 491.9; ICD-10-CM: J41, J42, J68.0), emphysema (ICD-9-CM: 492.0, 492.8; ICD-10-CM: J43, J98), or chronic obstructive pulmonary disease (COPD; ICD-9-CM: 491.20, 491.21, 491.22, 496; ICD-10-CM: J44.9) were excluded.

Supplemental Table 2. Visits and antibiotic prescriptions for acute respiratory illnesses by outpatient setting and antibiotic-indication tier, MarketScan Commercial Dataset, 2011 and 2018

	2011		2018		Comparison of 2011 and 2018		
**Setting**	**No. visits per 1000 enrollees (%)[A]**	**No. visits with antibiotic prescriptions per 1000 enrollees[B] (%)**	**No. visits per 1000 enrollees (%)[A]**	**No. visits with antibiotic prescriptions per 1000 enrollees[B] (%)**	**p-value for chi-square tests for proportion of visits with antibiotic prescription[C]**	**Prevalence rate ratio for no. visits per 1000 enrollees (99.99% CI)[D]**	**Prevalence rate ratio for no. visits with antibiotic prescriptions per 1000 enrollees (99.99% CI)[D]**
**All ARIs**							
Office	723 (87.4)	286 (87.6)	614 (80.9)	216 (78.6)	<.0001	0.85 (0.85-0.85)	0.76 (0.76-0.76)
Outpatient hospital	22 (2.6)	6 (1.8)	19 (2.6)	4 (1.5)	<.0001	0.90 (0.89-0.90)	0.72 (0.71-0.73)
Emergency department	5 (0.6)	2 (0.7)	6 (0.8)	2 (0.8)	<.0001	1.21 (1.19-1.23)	0.91 (0.89-0.93)
Urgent care	16 (1.9)	10 (3.1)	62 (8.2)	35 (12.7)	<.0001	3.97 (3.95-4.00)	3.52 (3.49-3.55)
Retail health	0 (0.0)	0 (0.0)	2 (0.2)	1 (0.3)	<.0001	272.21 (209.98-352.87)	198.81 (145.57-271.53)
Multiple	54 (6.5)	21 (6.6)	47 (6.2)	16 (5.7)	<.0001	0.88 (0.87-0.88)	0.73 (0.73-0.74)
Other[E]	8 (1.0)	1 (0.3)	8 (1.0)	1 (0.3)	<.0001	0.99 (0.98-1.00)	1.15 (1.11-1.19)
**ARIs for which antibiotics are almost always indicated**							
Office	12 (57.5)	6 (64.1)	10 (54.1)	6 (60.9)	<.0001	0.87 (0.86-0.88)	0.95 (0.94-0.97)
Outpatient hospital	2 (7.5)	0 (3.7)	1 (6.7)	0 (3.4)	<.0001	0.83 (0.80-0.85)	0.92 (0.87-0.98)
Emergency department	0 (0.9)	0 (1.1)	0 (1.5)	0 (1.7)	<.0001	1.46 (1.36-1.56)	1.62 (1.47-1.78)

	2011		2018		Comparison of 2011 and 2018		
Setting	No. visits per 1000 enrollees (%)[A]	No. visits with antibiotic prescriptions per 1000 enrollees[B] (%)	No. visits per 1000 enrollees (%)[A]	No. visits with antibiotic prescriptions per 1000 enrollees[B] (%)	p-value for chi-square tests for proportion of visits with antibiotic prescrip-tion[C]	Prevalence rate ratio for no. visits per 1000 enrollees (99.99% CI)[D]	Prevalence rate ratio for no. visits with antibiotic prescriptions per 1000 enrollees (99.99% CI)[D]
Urgent care	0 (1.3)	0 (2.1)	1 (5.0)	1 (8.0)	<.0001	3.50 (3.33-3.68)	3.93 (3.71-4.17)
Retail health	0 (0.0)	0 (0.0)	0 (0.1)	0 (0.2)	0.6257	291.98 (18.51-4605.71)	260.92 (16.53-4119.31)
Multiple	5 (23.2)	3 (27.7)	4 (22.4)	2 (24.1)	0.0062	0.89 (0.88-0.91)	0.87 (0.85-0.89)
Other[E]	2 (9.6)	0 (1.3)	2 (10.3)	0 (1.7)	<.0001	0.99 (0.97-1.02)	1.33 (1.21-1.46)
**ARIs for which antibiotics are sometimes indicated**							
Office	310 (85.6)	198 (88.5)	260 (78.3)	162 (80.1)	<.0001	0.84 (0.84-0.84)	0.82 (0.82-0.82)
Outpatient hospital	10 (2.8)	4 (1.8)	8 (2.5)	3 (1.4)	<.0001	0.81 (0.80-0.82)	0.73 (0.72-0.75)
Emergency department	3 (0.8)	2 (0.7)	3 (0.8)	1 (0.7)	<.0001	0.94 (0.92-0.96)	0.86 (0.83-0.88)
Urgent Care	10 (2.7)	7 (3.1)	36 (10.7)	25 (12.4)	<.0001	3.69 (3.66-3.72)	3.68 (3.65-3.72)
Retail health	0 (0.0)	0 (0.0)	1 (0.4)	1 (0.4)	0.0114	233.97 (173.59-315.35)	206.70 (147.33-290.00)
Multiple	28 (7.7)	13 (5.8)	22 (6.7)	10 (4.8)	<.0001	0.80 (0.80-0.81)	0.74 (0.74-0.75)
Other[E]	2 (0.4)	0 (0.2)	2 (0.7)	1 (0.3)	<.0001	1.45 (1.41-1.48)	1.25 (1.19-1.31)
**Antibiotic-inappropriate ARIs**							
Office	401 (90.2)	82 (88.0)	343 (84.3)	48 (76.5)	<.0001	0.85 (0.85-0.86)	0.59 (0.59-0.59)
Outpatient hospital	10 (2.2)	2 (1.7)	10 (2.4)	1 (1.6)	<.0001	1.00 (0.99-1.01)	0.64 (0.62-0.66)

	2011		2018		Comparison of 2011 and 2018		
**Setting**	**No. visits per 1000 enrollees (%)[A]**	**No. visits with antibiotic prescriptions per 1000 enrollees[B] (%)**	**No. visits per 1000 enrollees (%)[A]**	**No. visits with antibiotic prescriptions per 1000 enrollees[B] (%)**	**p-value for chi-square tests for proportion of visits with antibiotic prescrip-tion[C]**	**Prevalence rate ratio for no. visits per 1000 enrollees (99.99% CI)[D]**	**Prevalence rate ratio for no. visits with antibiotic prescriptions per 1000 enrollees (99.99% CI)[D]**
Emergency department	2 (0.5)	1 (0.6)	3 (0.8)	1 (0.9)	<.0001	1.53 (1.50-1.57)	0.93 (0.89-0.98)
Urgent care	6 (1.3)	3 (3.2)	26 (6.4)	9 (14.4)	<.0001	4.47 (4.42-4.51)	3.11 (3.06-3.15)
Retail health	0 (0.0)	0 (0.0)	1 (0.2)	0 (0.2)	<.0001	394.83 (231.13-674.47)	145.50 (63.20-334.97)
Multiple	21 (4.8)	6 (6.4)	21 (5.1)	4 (6.1)	<.0001	0.97 (0.96-0.97)	0.65 (0.64-0.66)
Other[E]	4 (1.0)	0 (0.2)	4 (0.9)	0 (0.3)	0.055	0.82 (0.81-0.84)	0.85 (0.79-0.92)

No. – Number; CI – Confidence interval; COPD – Chronic Obstructive Pulmonary Disease

[A] No. visits per 1000 enrollees calculated as no. visits/average no. enrollees x 1000. Values of 0 indicate <500 visits per 1000 enrollees.Percents may not sum to 100 due to rounding.

[B] No. visits with antibiotic prescriptions per 1000 enrollees calculated as no. visits with an associated oral antibiotic prescription within a 4-day post-visit window/average no. enrollees x 1000. Percents may not sum to 100 due to rounding.

[C] P-value for chi-square test comparing percent of visits with an antibiotic dispensed or administered in 2011 versus 2018, alpha = 0.0001.

[D] Referent is 2011, therefore a prevalence rate ratio of 0.80 indicates that the rate in 2018 was 20% lower than the rate in 2011.

[E] Other includes telehealth, schools, homeless shelters, Indian Health Services facilities, Tribal facilities, correctional facilities, patient homes, group homes, assisted living facilities, worksites, mobile healthcare units, birthing centers, military treatment facilities, custodial care facilities, hospice, adult living facilities, intermediate care facilities, psychiatric facilities, mental health centers, substance abuse facilities, rehabilitation facilities, dialysis facilities, ambulatory surgery centers, skilled nursing homes, long-term care facilities, inpatient hospital (outpatient services only) and outpatient not elsewhere classified.

Supplemental Table 3. Antibiotic prescriptions associated with visits for acute respiratory illnesses by antibiotic class, 2011 and 2018 MarketScan Commercial Database

Oral Antibiotic Class[A]	No. visits with antibiotic prescriptions per 1000 enrollees[B] (%) 2011	2018	Prevalence rate ratio comparing no. visits with antibiotic prescriptions per 1000 enrollees in 2011 versus 2018 (99.99% CI)[C]
**All ages**			
Penicillins	123 (37.9)	134 (48.6)	1.08 (1.08-1.09)
Macrolides	119 (36.6)	74 (26.8)	0.62 (0.62-0.62)
Cephalosporins	49 (15.0)	38 (13.6)	0.77 (0.77-0.77)
Fluoroquinolones	19 (5.9)	10 (3.5)	0.49 (0.49-0.50)
Tetracyclines	6 (1.8)	14 (5.1)	2.32 (2.29-2.34)
Sulfonamides	6 (1.8)	3 (1.1)	0.50 (0.49-0.51)
Other and multiple antibiotics	3 (1.0)	4 (1.4)	1.11 (1.09-1.13)

[A] Excludes prescriptions with missing antibiotic class data. Other includes antibiotics classified in MarketScan as urinary anti-infectives, other antibiotics, and other anti-infectives.

[B] No. visits with antibiotic prescriptions per 1000 enrollees calculated as no. visits with an associated oral antibiotic prescription within a 4-day post-visit window/average no. enrollees x 1000. Percents may not sum to 100 due to rounding.

[C] Referent is 2011, therefore a prevalence rate ratio of 0.80 indicates that the rate in 2018 was 20% lower than the rate in 2011.

[D] MarketScan Commercial datasets contain data on individuals aged <65 years. Calculated based on median age during MarketScan enrollment in each year.

Supplemental Table 4. Visits and antibiotic prescriptions for acute respiratory illnesses by region, MarketScan Commercial Dataset, 2011 and 2018

	Percent of visits with antibiotic prescription (99.99% CI)[A]		Comparison of 2011 versus 2018		
**Region**	**2011**	**2018**	**p-value for chi-square tests for proportion of visits with antibiotic prescription[B]**	**Prevalence rate ratio for no. visits per 1000 enrollees (99.99% CI)[C]**	**Prevalence rate ratio for no. visits with antibiotic prescriptions per 1000 enrollees (99.99% CI)[C]**
Northeast	37.1 (37.0-37.2)	33.8 (33.7-33.9)	<0.0001	0.93 (0.92-0.93)	0.84 (0.84-0.85)
Midwest	40.7 (40.6-40.7)	36.0 (35.9-36.1)	<0.0001	0.85 (0.85-0.86)	0.76 (0.76-0.76)
South	40.2 (40.1-40.2)	38.4 (38.4-38.5)	<0.0001	0.95 (0.95-0.95)	0.91 (0.91-0.91)
West	36.6 (36.5-36.7)	31.5 (31.3-31.6)	<0.0001	0.85 (0.85-0.86)	0.73 (0.73-0.74)

No. – Number; CI – Confidence interval; COPD – Chronic Obstructive Pulmonary Disease

[A] 99.99% CI estimated using a binomial distribution.

[B] P-value for chi-square test comparing percent of visits with an antibiotic dispensed or administered in 2011 versus 2018, alpha = 0.0001.

[C] Referent is 2011, therefore a prevalence rate ratio of 0.80 indicates that the rate in 2018 was 20% lower than the rate in 2011.
